# Spontaneous Intake and Long-Term Effects of Essential Oils After a Negative Postnatal Experience in Chicks

**DOI:** 10.3389/fvets.2019.00072

**Published:** 2019-03-15

**Authors:** Laurence A. Guilloteau, Anne Collin, Alexia Koch, Christine Leterrier

**Affiliations:** ^1^BOA, INRA, Université de Tours, Nouzilly, France; ^2^PRC, CNRS, IFCE, INRA, Université de Tours, Nouzilly, France

**Keywords:** essential oil, self-medication, broiler, chicks, postnatal experience

## Abstract

The postnatal period is critical for broiler chicks as they are exposed to potentially stressful environmental changes in the hatchery and during transportation to the rearing houses. The ability of broiler chicks to spontaneously drink essential oils (EO) to mitigate the effects of a negative postnatal experience was tested. Chicks were placed in the rearing facility either immediately (C group), or after a 24 h-delay period (D group) to mimic a delay in transportation possible under commercial conditions. In experiment 1, each group had access to either water only or to water and one EO (cardamom, marjoram, or verbena) from D1 to D13. Verbena EO intake was higher in the D group than in the C group from D1 to D6 and cardamom EO intake was lower in the D group than in the C group from D6 to D13. In experiment 2, half of the groups had access to water only and the other half had both water and the three EO simultaneously. Chicks from D and C groups chose the EO similarly except for cardamom EO with a lower intake being observed in the D than in the C group from D6 to D12. The delayed placement of the D group reduced chicken growth until 34 days of age and temporarily increased the feed conversion ratio, but did not affect their welfare or the prevalence of health disorders. The EO intake did not mitigate the growth reduction in D group chicks, but did mitigate the reduced *Pectoralis major* muscle yield. In conclusion, chicks were able to make spontaneous choices regarding EO intake according to their postnatal experience when EO were presented individually, but not when presented simultaneously as in our experimental design. The EO intake only partially mitigated the decrease in chicken performance after the negative postnatal experience.

## Introduction

The postnatal period is a critical period for livestock. They have to cope with specific husbandry conditions, and exposure to adverse environmental conditions that may result in stress responses. Stress during early life can induce persistent changes in physiology, behavior, and immune phenotype (1). Strengthening an animal's robustness, that is to say its capacity to adapt to environmental disturbances, during the postnatal period is a potential strategy to reduce the immediate, and long-lasting effects of stressful early experiences. It can also contribute to improving the animal's sanitary status and to reducing the use of antimicrobial drugs. One approach initially observed in wild animals is the stimulation of self-medication behavior (SM) or zoopharmacognosy. This has been defined as the ability of animals to select and use specific plants or substrates with medicinal properties to control or to prevent diseases (2) or situations of discomfort. Forbey et al. (3) defined self-medication (as homeostatic behavior. In farm animals, observations of SM have been reported in ruminants (4) and research has mainly focused on plants associated with anti-parasitic properties (5, 6).

In order to reduce the use of chemical antimicrobial drugs in farm animals alternative solutions to these medicines are encouraged (7). Essential oils (EO) extracted from medicinal plants have multi-functional properties including antimicrobial, antioxidant, immunostimulatory, anti-inflammatory, and nervous system regulatory properties (8–11). These properties are related to the composition of the EO, which are mainly terpenoids (monoterpenes, sesquiterpenes) and a variety of aromatic compounds. Phenols, alcohols, ketones, and aldehydes are the molecules usually associated with antibacterial action (12). Phenylpropanoids (13) and terpenoids such as the oxide 1,8-cineole are known to have anti-inflammatory properties, and positive effects on the digestive, and respiratory systems (14, 15).

In chickens, EO have been investigated as growth and health promoters and have been used as feed additives (15–19). In these studies, EO were included in feed and therefore chickens had no choice but to ingest them. If chickens were able to select EO with medicinal effects that were the most adapted to a challenging situation, their robustness would potentially be improved and drug use reduced.

To test the hypothesis that chicks are able to spontaneously consume EO in response to the needs induced by their experience, we developed an experimental setting which reproduced a negative experience highlighted in previous studies. These studies reproduced the adverse conditions chicks are subjected to during the postnatal period. In poultry production systems, chicks are transported from the hatchery to rearing houses and are subjected to stressors such as temperature variations, jolts in transportation boxes due to truck movements, and feed and water deprivation lasting between several hours and 2 or 3 days after hatching. Deprivation of feed and water in chicks has long-lasting effects on performance (20–22) and also on physiological and immune parameters (21, 23), which can result in greater susceptibility to diseases and mortality (23). The long-lasting effects of post-hatch transportation have also been described in terms of chick behavior, health, and performance (24–28). The term “negative experience” was used to qualify the postnatal treatment experienced by chicks whether it resulted in long lasting effects or not.

In this study, two experiments were performed. The first was designed to assess the capacity of chicks to spontaneously choose to ingest EO and to analyze whether this intake was related to their postnatal experience. The second aimed to assess the capacity of chicks to choose between three EO in addition to drinking water and also observe the kinetics of EO choice, and analyze the effects of EO on chicken performance, welfare, and health s over the whole growing period.

## Materials and Methods

All procedures used in these experiments were approved by the local ethics committee (Comité d'Ethique en Expérimentation Animale Val de Loire, Tours, France; permission no. 01730.02 and 2015070815347034v2, APAFIS#1082) and carried out in accordance with current European legislation (EU Directive 2010/63/EU).

### Model of a Postnatal Negative Experience in Chicks

After hatching, chick transportation to the broiler farms can occur under suboptimal conditions (24–28). To analyze the consequences of this experience over the whole growing period, eggs (Hubbard Classic®, Quintin, France) were incubated for 21 days under standard conditions. After opening the incubator (T0), the chicks were sex-sorted according to their plumage, wing-tagged, and vaccinated against infectious bronchitis (IB) (NOBILIS IB 4/91®, Intervet, Beaucouzé, France) by the conjunctival route. The chicks were then either placed immediately in pens in the rearing facility after their removal from the incubator (Control group, C) or were removed and placed in transportation boxes for a period of 24 h before their placement (Delayed group, D). The latter group were deprived of feed and water and subjected to irregular movement and variable room temperatures: 32°C (30 min), 21°C (90 min), 32°C (30 min), and then at 21°C with alternating cycles of box movement (M) and immobility (I) for 24 h. One cycle consisted of 45 min (M), 15 min (I), 30 min (M), and 30 min (I). These conditions were combined to be the closest to the actual suboptimal conditions experienced by broiler chickens. Chicks were allotted to each of the two groups according to the time of hatching [50% that hatched in the incubator more than 12 h before T0, and 50% that hatched between 12 and 0 h (= T0) and sex with 50% male/50% female as determined at T0]. Chicks were reared at the Experimental animal center of Tours (PEAT) (INRA Center Val de Loire, France) under standard temperature and light conditions with *ad libitum* access to water and with a wire mesh platform and a perch for environmental enrichment. At D13, the chickens were transferred to another livestock building for the growth phase until D34. They had *ad libitum* access to feed without anticoccidial drugs. They were fed with a standard starting diet (metabolizable energy = 12.8 MJ/kg, crude protein = 22%) until 19 days and then a rearing diet from 19 to 34 days.

### Essential Oils

The essential oils (EO) were chosen for their recognized complementary properties to control infectious challenges, reduce stress response, and improve digestive and immune system functions. Three EO were chosen based on the scientific literature, expert advice from practitioners, and preliminary results from experiments performed with 12 EO in chickens. Cardamom (*Elettaria cardamomum*) (1480CQ, batch S12A, Herbes et Traditions, Comines, France), marjoram (*Origanum majorana*) CT thujanol (2507CQ, batch S12D, Herbes et Traditions), and lemon verbena (*Lippia citriodora*) (FLE094, batch H181013MA, Florihana, Caussols, France) were used to assess the spontaneous intake of EO in the C and D groups. In addition to their antimicrobial and antioxidant activities (29–31), these EO have complementary properties. Cardamom EO has been demonstrated to have antispasmodic and anti-inflammatory activities (32), and gastroprotective properties (33). Marjoram EO has a variety of biological activities, including a hepatoprotective role (31). Lemon verbena EO has been shown to have analgesic, anti-inflammatory, sedative, and digestive properties (34).

Based on previous studies (35), each EO was diluted in water (0.001%), mixed and shaken vigorously before being made available in a drinking bottle. The main components obtained by gas chromatography coupled to mass spectrometry for each EO are listed in Table 1.

**Table 1:** Composition of essential oils.

**Compound**	**Cardamom** ***Elettaria cardamomum***		**Marjoram** ***Origanum majorana*** **CT thujanol**		**Verbena** ***Lippia citriodora***	
	**Specification** **(%)**	**Relative content (%)^a^**	**Specification** **(%)**	**Relative content (%)^b^**	**Specification** **(%)**	**Relative content (%)^c^**
Monoterpenes	6–12	13	30	40	5–15	29
Sesquiterpenes				3	18–26	24.5
Monoterpenols	3–6	5	40–50 (20 thujanol)	50 (25 thujanol)	3–15	2
Esters	39–51	36		2		
Oxides	27–35	34			<7	5
Aldehydes					20–40	24

a *1480CQ, batch S12A, Herbes et Traditions, Comines, France*.

b *2507CQ, batch S12D, Herbes et Traditions*.

c *FLE094, batch H181013MA, Florihana, Caussols, France*.

### Experimental Design

#### Experiment 1

**Figure 1:**
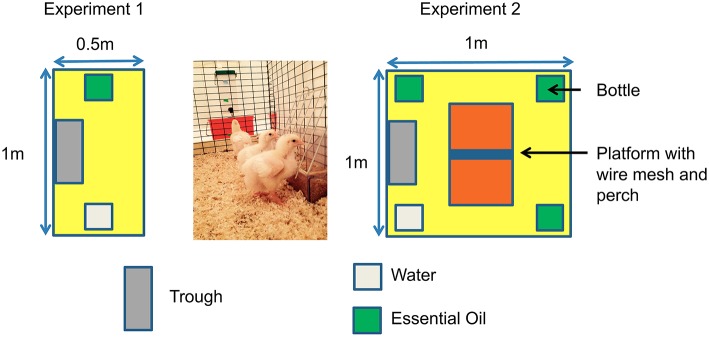
Experimental pen organization.

After opening the incubator, 192 chicks were placed in pens (0.5 × 1 m) and allocated to either C group (*n* = 96) or D group (*n* = 96). Chicks were provided with feed and water (W) *ad libitum* in the pens from D0 to D13 for the C group and from D1 until D13 post-hatching for the D group. Chicks were allocated either to pens with water only (W groups) or to pens with water and one essential oil (3 EO groups), cardamom, marjoram, or verbena, i.e., four groups each for C and D placement conditions, giving a total of eight groups (six pens/group, four chicks per pen) (Table 2). Each essential oil (EO) was placed at D1 in the EO-C and EO-D groups. Two bottles, one of water and the other containing one of the EO were available in each pen for the EO groups. Two bottles of water were available for the W groups (Figure 1). The bottle position was changed every day for a week and every 2–3 days during the second week to prevent the chicks from getting used to the position of the bottles. The intake of water and of each EO was recorded at D1, D2, D4, D6, D9, and D13. Water and EO were changed each time intakes were recorded and filled up between two intake measures if necessary. Water and EO intakes were first compared between groups for the 13 days post hatching when EO were provided. Water and EO intakes were then expressed as a percentage of the total liquid intake since differences in this total were observed between C and D groups.

**Table 2:** Experimental design.

**Hatching condition**	**Control group**		**Delayed group**	
**Oil provision**	**No**	**Yes**	**No**	**Yes**
**Group name**	**W-C**	**EO-C**	**W-D**	**EO-D**
**EXPERIMENT 1**				
Water	24 (4 × 6)^*^		24 (4 × 6)	
Cardamom EO		24 (4 × 6)		24 (4 × 6)
Marjoram EO		24 (4 × 6)		24 (4 × 6)
Verbena EO		24 (4 × 6)		24 (4 × 6)
**EXPERIMENT 2**				
Water	96 (16 × 6)		96 (16 × 6)	
EO provision^†^		96 (16 × 6)		96 (16 × 6)

* *(number of chicks × number of pens)*.

† *EO provision = cardamom EO and marjoram EO and verbena EO*.

#### Experiment 2

After opening the incubator, 384 broiler chicks were either placed in pens (1 × 1 m, 16 chicks/pen) immediately in the C group (*n* = 192) or after 24 h of negative experience in the D group (*n* = 192). Before being placed in pens, half of the chicks (*n* = 192) were randomly chosen to be examined macroscopically to determine their quality scores as proposed by Tona et al. (36). Only criteria focusing on the retracted yolk [non-retracted: 0, retracted:12 (23% of total score)], navel area [not closed and discolored:0; not completely closed and not discolored: 6; completely closed and clean: 12 (23% of total score)], remaining membrane [very large membrane:0; large membrane: 4; small membrane: 8; no membrane: 12 (23% of total score)] and remaining yolk around the navel area [very large yolk: 0; large yolk: 8; small yolk: 12; no yolk: 16 (31% of total score)] were considered to establish a total score reported to 100%. Chicks were allocated to the W-C or W-D, or EO-C or EO-D group (six pens/group, 16 chicks/pen). Besides feed and water supplies, *ad libitum* access to the three EO was provided separately in EO groups (EO-C or EO-D) from D1 until D12 post-hatching. One bottle containing water, and three others each containing one of the EO, were placed in each pen (Figure 1). The other half of the chicks (six pens each for C and D groups) only had access to water in four bottles (W-C and W-D groups). As in the first experiment, the bottle position was changed every day for a week and every 2–3 days for the second week. At D13, the chickens were transferred to another livestock building, placed in larger pens (2 × 1 m) and reared under standard conditions without access to EO. The intake of water and of each EO was recorded at D1, D2, D4, D6, D9, and D12. Water and EO were changed each time intake was recorded and supplemented if necessary between two measures. The EO intake was expressed as the percentage of EO intake in relation to the total liquid intake.

### Performance Measurements

Body weight was measured at D0, D6, D9, and D13 in experiment 1, and at D0, D1, D6, D12, D19, D27, and D33 in experiment 2. Feed consumption was measured in each pen for the periods between D1-D6, D6-D9, and D9-D13 in experiment 1, and between D0–D6, D6–D12, D12–D19, D19–D27, and D27–D33 in experiment 2, and then used to calculate the feed conversion ratio (FCR). Twelve chickens per group (two/pen) were necropsied at D1 and D13 to measure the weight of the yolk sac and at D34 to measure the weight of the *P. major* muscle (experiment 2).

### Welfare Status Assessment (Experiment 2)

Several tests were used to measure fearfulness since reactivity has been observed to increased when birds experience stressful situations and this is why some of these tests are included in the Welfare Quality® protocol (37).

#### Tonic Immobility Test

Tonic immobility is a behavioral response modulated by frightening situations and its duration is considered to measure the level of fearfulness (38). Tonic immobility was induced by restraining the animal on its back: the longer the bird needed to right itself (referred as TI), the more fearful the bird was. Four 7-day-old chicks per pen were placed on their back in a *U*-shaped cradle and restrained for 10 s and the duration of tonic immobility was recorded. If a chick failed to right itself after 5 min, a maximum score of 300 s was recorded. If tonic immobility was not induced after five attempts, a score of 0 s was recorded.

#### Novel Object Test

A novel object test was used to assess bird reactions to novelty with a protocol adapted from the Welfare Quality® protocol. The novel object used was a 50 cm long and 3-cm wide stick with colored bands. Five minute after entering the pen, the observer placed the novel object on the litter between the trough and the bottles. The observer moved back 1.5 m, remained standing, and counted the number of chicks at a distance of <1 chick length from the object and the number of chicks that pecked the object every 30 s for a total of 2 min. This sampling was performed in each pen at 22 days of age.

#### Reaction-To-Human Test

The avoidance distance test described in the Welfare Quality® protocol was adapted to our experimental room to assess the human-animal relationship. The observer entered the pen and remained standing close to the door due to the small size of the pens which did not allow walking without greatly disturbing the chickens. The duration that was needed for at least three chickens (*n* = 12/pen) to come close to the observer (<1 m away) was recorded. This test lasted 2 min and was performed in each pen at 23 days of age.

### Health Status Assessment (Experiment 2)

General health status and the possible presence of respiratory and digestive disorders, and tarsal angulations were assessed by a veterinarian through a clinical inspection or auscultation and was recorded each time that body weight was recorded. There was no evidence of hock burn or of foot pad dermatitis, so only lameness was measured on D29 using the Welfare Quality® gait scoring method. The identity numbers of chickens scored for gait analysis were randomly chosen (two males and two females/pen) before assessment. Gait scoring was performed by experts on four chickens per pen using a score from 0 (normal gait) to 4 (severe abnormality, only able to walk a few steps).

Immune system activity was assessed by measuring the antibody titers specific to the infectious bronchitis (IB) vaccine that were present in the serum of the chicks at hatching (*n* = 20) and at D13 (*n* = 20/group) and D34 (*n* = 20/group) after vaccination (Experiment 2). Antibody titers were determined by ELISA using the ID Screen® IBV Indirect kit and the protocol described by the supplier (ID.vet, Grabels, France).

### Statistical Analysis

Analyses were carried out using XLSTAT software (version 2015, Addinsoft, Paris, France). The effects of the delayed placement and EO supply on total liquid intake, water intake, total EO intakes, body and muscle weight, and the FCR ratio were analyzed by ANOVA after having checked the normality of residuals distribution and the homogeneity of variances. The fixed effect model was yij = μ + Di + EOj+ (D-EO), where Di, EOj and (D-EO)ij, were the fixed effects of the Delayed placement, EO provision, and (D-EO)ij the interaction of Delayed placement with EO provision. When there were interactions between variables, the Fisher (LSD) test was used to determine the significant differences between groups. Because the residuals were not normally distributed and variances were not homogenous between groups, data on each EO intake, behavioral tests, and gait scores were analyzed with non-parametric tests: the Kruskal-Wallis test for the EO effect and the Mann-Whitney test for the comparison between the D and C groups for each period. The effects of periods on EO intake were analyzed with the non-parametric Friedman test. The Dunn test with the Bonferonni correction was used as a *post-hoc* test after Kruskall-Wallis and Friedman analyses. The clinical data and quality score of the chicks were analyzed using a Chi-squared test.

Differences were considered to be significant when *p*-values were below 0.05, to be a tendency when *p*-values were between 0.05 and 0.1 and not significant (NS) when *p*-values were above 0.1. The values are presented as means ± standard deviations or medians, quartiles, maximum, and minimum values.

## Results

### Spontaneous Intake of One EO by Chicks After a Negative Postnatal Experience (Experiment 1)

The chicks drank significantly fewer liquids (water and EO) in the D group than in the C group whether the group had access to water only or both water and EO, but there was no EO effect within D or C groups (Table 3). Chicks in both D and C groups drank less water in the EO groups than in W groups and within these groups, they drank less water in the D than in the C groups (Table 3). To overcome the effect of the liquid intake difference between D and C groups, the intake of EO was then normalized by reporting the intake of EO to the intake of liquids during each period analyzed. The term “intake” used thereafter corresponds to this ratio.

**Table 3:** Liquid intakes in each group according to the treatment (Experiment 1).

	Total intake^*^		Water intake^*^		Oil intake^*^	
**Treatment**	**Control**	**Delayed**	**Control**	**Delayed**	**Control**	**Delayed**
**OIL GROUP**						
Water only	808 ± 114	674 ± 42	808 ± 114^b^^†^	674 ± 42		
Verbena	739 ± 26	624 ± 44	588 ± 66^a^	441 ± 168	151 ± 81	183 ± 146
Cardamom	747 ± 66	613 ± 43	521 ± 139^a^	453 ± 123	226 ± 154	134 ± 106
Marjoram	719 ± 94	638 ± 37	614 ± 77^a^	442 ± 111	106 ± 77	196 ± 106
**ANOVA**						
Treatment	*F*_(1,40)_ = 33.6; *p* < 0.0001		*F*_(1,40)_ = 15.2; *p* < 0.001		*F*_(1,42)_ = 0.1; NS	
Oil	*F*_(3,40)_ = 2.5; *p* = 0.084		*F*_(3,40)_ = 12.8; *p* < 0.0001		*F*_(2,42)_ = 0.2; NS	
Interaction	*F*_(3,40)_ = 0.4; NS		*F*_(3,40)_ = 0.4; NS		*F*_(2,42)_ = 1.7; NS	

* *Each intake (mL) represents the intake mean (m ± sd) per animal from 5 to 6 pens of the same group*.

† *Different superscript letters indicate significant differences in water intake between oil groups whatever the treatment*.

*The p-values indicate significant differences between the control and delayed groups*.

**Figure 2:**
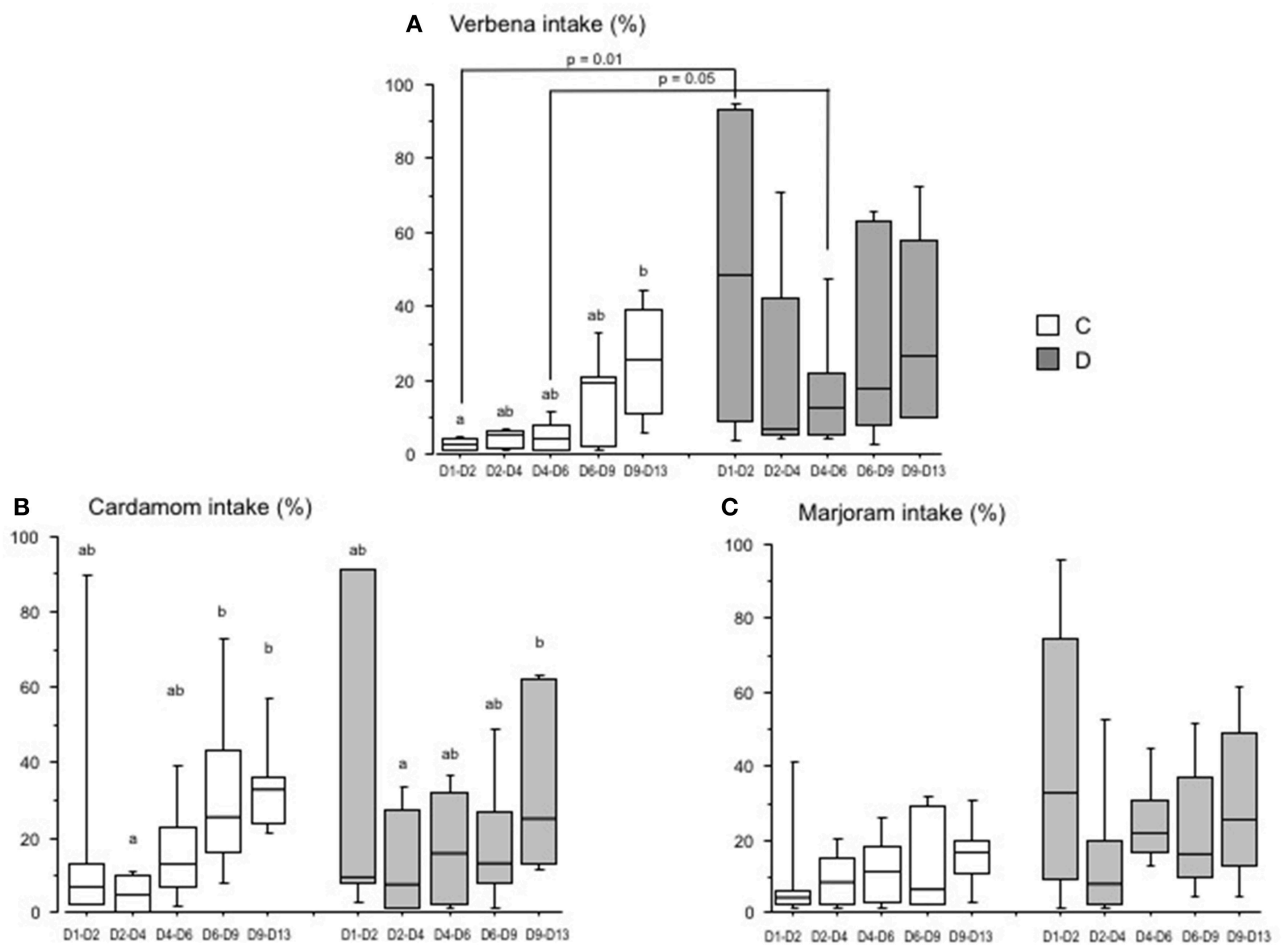
Essential oil intake by chicks over time (Experiment 1). Chicks were either directly placed in pens (C group; white) or delayed for 24 h (D group; gray). The histograms show the box-plots and whiskers of EO intake (EO/(water + EO), %) for each group, verbena **(A)**, cardamom **(B)**, and marjoram **(C)**. Different letters indicate significant differences in EO intake between periods of measurement for each group of chicks (Dunn test). The *p*-values indicate significant differences between the C and D groups within each period (Mann-Whitney test).

There was a high variation in intake of the different EO between pens and for both groups of chicks (C and D groups). However, there was a significant progressive increase in the EO intake over time for some EO (Figure 2). The intake of verbena EO was significantly higher for the period D9 to D13 compared to D1–D2 in the C group (Figure 2A). In contrast, there were no significant differences in the intake of verbena EO for the D group during the period D1 to D13. For the C group, the intake of cardamom EO was higher for the period D6 to D13 than that for the period D2–D4 (Figure 2B). For the D group, the intake of cardamom EO was only higher for the period D9 to D13 compared to D2–D4. There were no significant differences over time in the intake of marjoram EO for either the C or D groups (Figure 2C).

EO intake differed according to the group's postnatal treatment. The intake of verbena EO was significantly higher in the D than in the C group for the periods D1–D2 and D4–D6, the amount of EO consumed was the highest between D1 and D2 in the D group (*p* = 0.01) (Figure 2A). The intake of cardamom EO was not significantly different between the D and C groups (Figure 2B). There was only a tendency for the D group to drink more marjoram EO than the C group between D1 and D6 (25.4 ± 14.4 vs. 10.9 ± 7.1 in the D group and C group, respectively, *p* = 0.1) (Figure 2C).

These results show that the chicks spontaneously and rapidly drank more verbena EO when their placement in the rearing facility was delayed than when they were placed directly after hatching.

### Choice and Spontaneous Intake of Simultaneously Presented EO by Chicks After a Negative Postnatal Experience (Experiment 2)

In this experiment, the chicks had the choice to drink any of the three EO used in the first experiment in addition to freely accessible water in their pen. As in the first experiment, the chicks consumed significantly fewer liquids (water and EO) in the D group than in the C group for the 12 days after hatching. They also drank less water in the EO groups than in the W groups independent of the postnatal treatment during this period (Table 4).

**Table 4:** Liquid intakes in each group according to the treatment (Experiment 2).

	Total intake^*^		Water intake^*^		Oil intake^*^	
**Treatment**	**Control**	**Delayed**	**Control**	**Delayed**	**Control**	**Delayed**
**OIL GROUP**						
Water only	518 ± 11	435 ± 9	518 ± 11	435 ± 9		
Oil combination	562 ± 91	482 ± 131	283 ± 87	252 ± 141	279 ± 16	230 ± 33
**ANOVA**						
Treatment	*F*_(1,20)_ = 83.2; *p* = 0.022		*F*_(1,20)_ = 2.8; NS		*F*_(1,22)_ = 10.6; *p* = 0.009	
Oil	*F*_(1,20)_ = 2.7; NS		*F*_(1,20)_ = 37.6; *p* < 0.0001		–	
Interaction	*F*_(1,20)_ = 0.1; NS		*F*_(1,20)_ = 0.6; NS		–	

* *Each intake (mL) represents the intake mean (m ± sd) per animal from 5 to 6 pens of the same group*.

*The p-values indicate significant differences between the control and delayed groups*.

**Figure 3:**
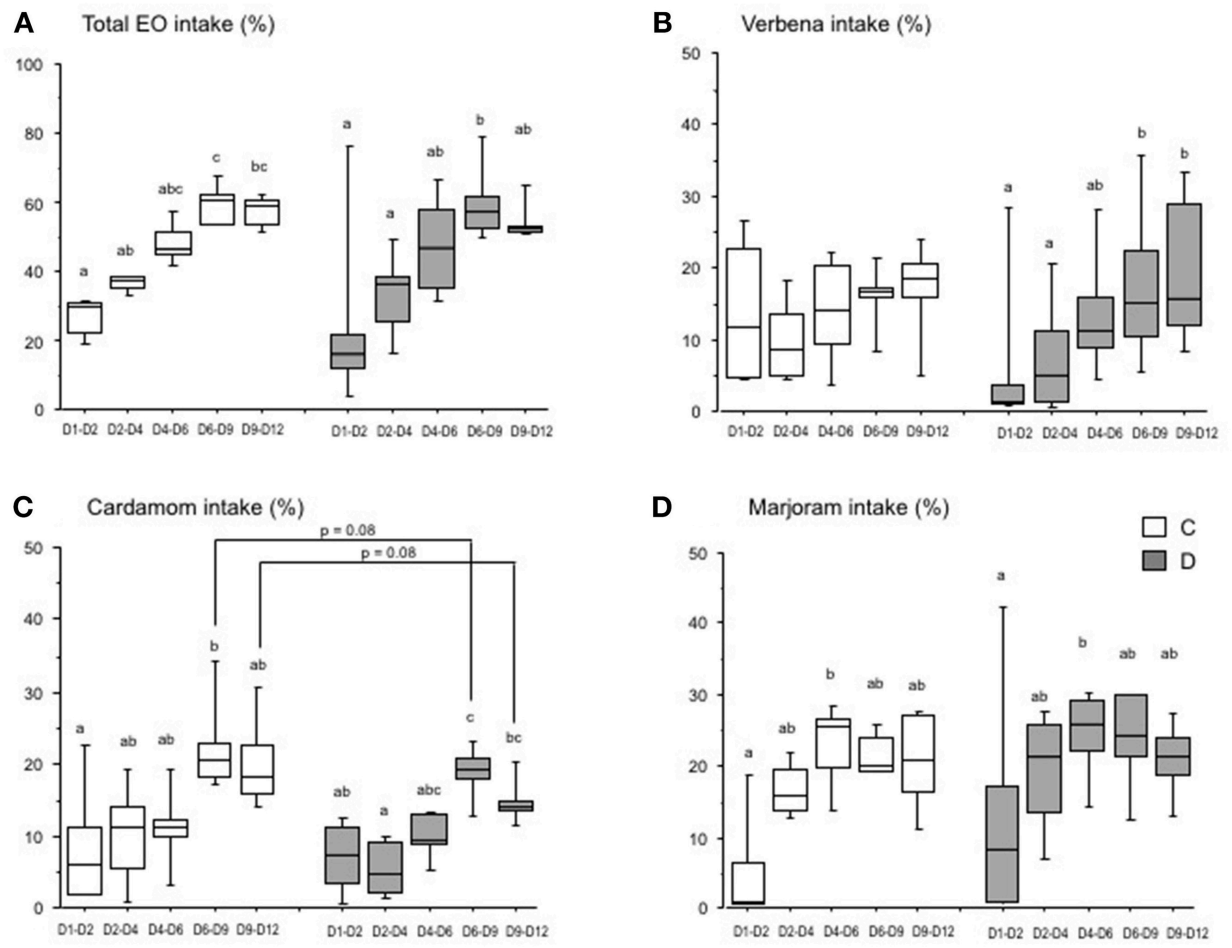
Essential oil intake by chicks over time (Experiment 2). Chicks were either directly placed in pens (C group, white) or delayed for 24 h (D group; gray). The histograms show the box-plots and whiskers of EO intake (EO/(water + EO), %) for each group, and the three EO **(A)**, verbena **(B)**, cardamom **(C)**, and marjoram **(D)**. Different letters indicate significant differences between periods for each group of chicks (Dunn test). The *p*-values indicate significant differences between the C and D groups within each period (Mann-Whitney test).

As in the first experiment, there was a large variation in intake of each EO between pens, and there was a significant progression in the intake of EO between D1 and D12 for both the C and D groups (Figure 3A). There were no significant differences in the intake of verbena EO by the C group over time, but the intake by the D group increased significantly from the D1–D2 to D6–D9 period (Figure 3B). The intake of cardamom EO by the chicks increased progressively and significantly between D1–D2 and D6–D9 for the C group and from D1–D4 to D6–D9 for the D group (Figure 3C). It was the same for the intake of marjoram EO from the period D1–D2 to D4–D6 for both groups of chicks (Figure 3D).

The spontaneous intake of EO differed between the three EO available in pens depending on the postnatal treatment. The intake of cardamom EO was significantly lower in the D group than in the C group from D6 to D12 after hatching (16.4 ± 3.0 in the D group vs. 21.1 ± 6.2 in the C group, *p* = 0.05), but only a tendency (*p* = 0.08) when comparisons were carried out between D6–D9 and D9–D12 (Figure 3C). However, the difference in intake of verbena and marjoram EO was not significant between the C and D groups whatever the intake period (Figures 3B,D).

These results show that when three EO were available simultaneously, the intake of EO by the chicks changed over time within each group of postnatal treatment, but EO were not differently chosen by chicks between groups except for the delayed chicks which drank less cardamom EO than control chicks.

### Effects of EO Intake on Chick Performance (Experiments 1 and 2)

In experiment 1, the delay of 24 h before placing the chicks in the D group significantly reduced the chicks' growth when they were transferred to the rearing building and until D13 (decrease in weight of 14.8% in the D group compared to the C group, *p* < 0.0001). The reduction in growth in the D group was not mitigated by EO intake (data not shown), but the FCR was significantly lower during the period of D6 to D9 in the EO groups (1.308 ± 0.119) than in the W groups (1.418 ± 0.22), independent of the postnatal treatment [*F*_(1, 44)_ = 4.5; *p* = 0.039].

In experiment 2, 162 chicks among the 192 examined at T0, before any treatment or placement, had an overall quality score above 36 points (out of 52 points, 70%) (class 1) and only 30 chicks had a score below 36(class 2). Chick weights at different times after hatching did not differ between class 1 and class 2.

**Figure 4:**
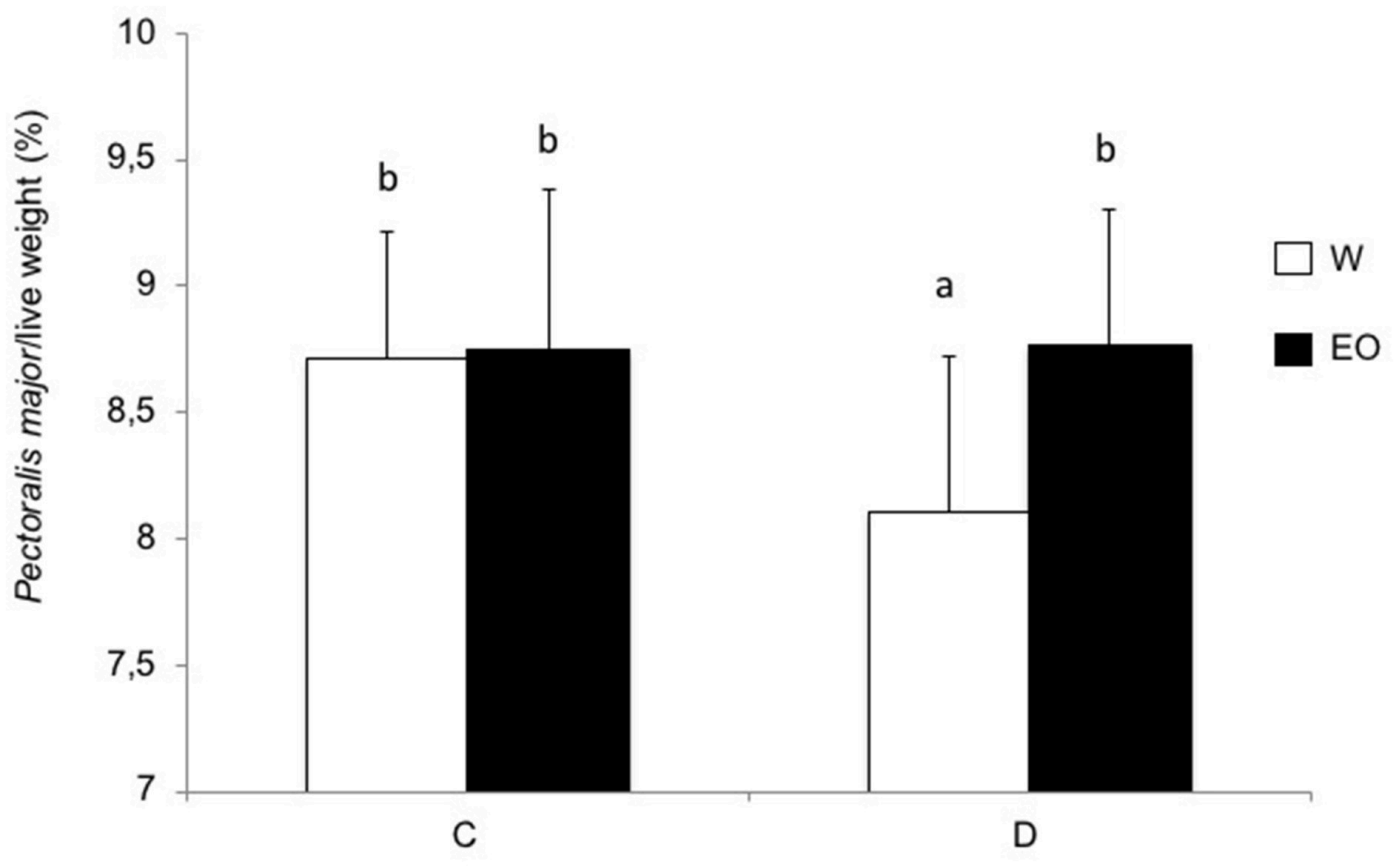
Chicken performance in experiment 2. Chicks were either directly placed in pens (C group) or delayed for 24 h (D group), and had *ad libitum* access to only water (W) or to water, and EO (EO). The histograms show *P. major* weight at 34 days of age for chickens that had *ad libitum* access to only water (W) or to water and EO (EO). The results express the mean and standard deviation. NS or *p*-values indicate statistical significance between the W and EO groups.

The size and the presence of vitellus in the chicks were not affected by the postnatal experience or EO ingestion at D13 and D34. Chicks in the D group showed a marked significant reduction in growth from D1 to D34 (6.5%, *p* < 0.0001) (data not shown). The FCR in the D group (1.5 ± 0.07) was significantly impaired [*F*_(1, 20)_ = 19.6] after a change in environment (building and feed changes) between D12 and D19 compared to the C group (1.39 ± 0.05). This was reversed during the following period D19–27 [1.43 ± 0.02 in the D group vs. 1.46 ± 0.02 in the C group; *F*_(1, 20)_ = 13.5; *p* = 0.002] and there was no longer a difference after D27. EO intake had no significant effect on chick growth or on FCR whatever the postnatal experience. However, at D34, the *P. major* muscle yield was significantly higher in the chickens that had access to EO (Figure 4), suggesting that EO intake had had a positive effect on the growth rate of *P. major*.

### Effects of EO Intake on Chick Welfare (Experiment 2)

Delayed placement and EO supply had no effect on tonic immobility duration (51.6 ± 40.4 in the C group vs. 56.8 ± 44.7 in the D group; 59.1 ± 45.1 in the W group vs. 49.2 ± 39.5 in the EO group; *p* > 0.1). The number of attempts needed to induce this behavior did not differ between groups (1.6 ± 0.8 in the C group vs. 1.7 ± 0.7 in the D group; 1.6 ± 0.7 in the W group vs. 1.7 ± 0.8 in the EO group; *p* > 0.1).

During the novel object test, the mean number of chickens close to the object or pecking at it in each group was not influenced by the delayed placement or by EO supply regardless of the scan period (0, 30, 60, 90, and 120 s). The average number of chickens close to the object was 3.7 ± 1.7 in the C group vs. 2.9 ± 1.5 in the D group (*p* > 0.1) and 3.5 ± 1.5 in the W group vs. 3.2 ± 1.9 in the EO group (*p* > 0.1). The maximal number of chickens pecking at the object was also similar between groups (0.9 ± 2.0 in the C group vs. 1.6 ± 2.2 in the D group; *p* > 0.1; and 1.4 ± 2.2 in the W group vs. 1.1 ± 2.1 in the EO group; *p* > 0.1).

Between 3 and 10 chickens per pen moved close to the observer in the reaction-to-human test. The mean number of chickens close to the observer over all scan periods was not influenced by delayed placement (4.8 ± 1.7 in the C group vs. 5.6 ± 1.5 in the D group; *p* > 0.1). The number of chickens close to the observer 60 s after starting the test was lower in the EO group (4.6 ± 1.6 in EO pens vs. 6.5 ± 2.0 in W pens, *p* = 0.03) and the mean number over all scan periods tended to be lower in the EO pens than in the W pens (4.5 ± 1.4 in EO pens vs. 5.9 ± 1.6 in W pens, *p* = 0.06).

### Effects of EO Intake on Chick Health (Experiment 2)

A slight respiratory impairment was present in 38.2% of chickens at D19 but no longer at D33. Diarrhea was present in 26.5% of chickens and severe abnormal angulations in 1.8% at D33. The prevalence of these disorders did not differ between the C and D groups, or between chicks that were in quality score class l at hatching (good quality) compared to class 2, or between chicks with or without access to EO. The gait score (2.21 ± 0.59) was not affected either by the negative postnatal experience or by EO supply. The global mortality rate in the experiment was 2.6%. It was associated with a lower chick quality score at hatching (37.3 ± 6.9 in dead animals vs. 45.1 ± 6.8, in other chicks *p* = 0.04). Chicks died either of heart attacks (*n* = 3) or were euthanized because of severe locomotor disorders (*n* = 5).

Regarding the reactivity of the immune system, the antibody response against IB vaccine dropped after hatching (10644 ± 1384 at D0 vs. 1173 ± 1038 at D34). The antibody titers tended to be higher in the D group (3926 ± 2553) than in the C group (2928 ± 1650) at D13 [*F*_(1, 66)_ = 2.8; *p* = 0.097] independently of access to EO, the titers did not differ between the C and the D groups or between chicks with or without access to EO at D34.

## Discussion

The present study investigated the capacity of chicks to select and consume EO after their exposure to a negative postnatal experience related to the delay between their hatching and transportation to the rearing facilities. The chicks consumed significantly fewer liquids (water and EO) in the delayed group than in the control group for 12 days after hatching, possibly linked to the significant reduction in body weight induced by delayed placement for the D group. Regarding the EO intake itself, a considerable variation was found in EO intake between the chick pens of the delayed group, particularly in the days following the stressful event (D1–D2) and it was also true for the control group to a lesser extent. A progressive increase in EO intake was observed over time for most of the EO. In the first experiment, when the chicks had the choice to consume water or one EO, the cardamom EO intake increased significantly over time and it started earlier for the C group (D6) than for the D group (D9). It was also the case for the intake of verbena EO in the C group (D9) but it was not the case for the D group or the marjoram EO for either group. Many animals can use medication by selecting and eating specific plants (39). The process involved in medication behavior is complex and the involvement of innate vs. learned behavior has been discussed regarding both therapeutic and prophylactic medication (40, 41). In general, the factors discussed have been restricted to immune defenses against parasites and the process of learning about food containing secondary plant compounds (40, 41). In our study, we chose to introduce EO in water and as a supplement to water available to differentiate between water intake for thirst and spontaneous intake of EO by chicks. The immediate intake of verbena EO by the D group could suggest an innate behavior of medication, whereas the progressive intake of this EO by the C group and of cardamom EO for both groups could suggest a learning process over time.

In the second experiment, when the three EO were simultaneously available, there was also a progressive increase in EO intake over time for both groups, except for verbena EO intake in the C group. This was in fact the opposite result to the first experiment, the verbena EO intake by the D group increased progressively over time, whereas the intake was immediate and constant for the C group. We can assume that it was more difficult for the chicks in the D group to learn from post-ingestion signals since these signals were probably confused because of the simultaneous provision of the three EO. It has been shown using diets with different energy levels that chicks are able to develop preferences when they have acquired experience of the post-ingestion cues of the diets (42). This suggests that for chick to choose between several EO they would need a previous experience with each EO separately. However, we do not have any explanation for the immediate and persistent intake of verbena EO by the C group in experiment 2.

When the EO were presented separately (experiment 1), the chicks spontaneously consumed verbena EO over the period from D1 to D13 and in significantly higher amounts in the D group than in the C group from D1 to D6. There was a tendency for the D group to consume more marjoram EO from D1 to D6 than the C group. These results show that chicks were able choose spontaneously to drink verbena EO, and possibly marjoram EO, immediately after their negative postnatal experience and for a week. During that period, the control group drank a small amount of these EO.

Several conditions have been identified to define the behavior of SM: (1) infection or discomfort induces SM behavior, (2) SM improves the fitness of infected animals, and (3) SM behavior is costly to non-infected animals (39, 43). In chickens, one study has reported the preference of lame chickens for a feed supplemented with an anti-inflammatory and analgesic drug (Carprofen) rather than the same feed without the drug (44). This study suggested that lame broilers found a benefit in eating feed supplemented with carprofen and may have selected carprofen for its analgesic properties. The control chickens tended to avoid feed supplemented with carprofen, suggesting an aversion to this drug. In our study, the high intake of verbena EO by the delayed chicks and its low intake by the control chicks during the 6 days after the negative postnatal experience suggest that delayed chicks may have selected verbena EO for its beneficial properties. The antioxidant, anti-inflammatory, sedative, and digestive effects of lemon verbena are well-reported in *in vitro* and *in vivo* studies and more recently the beneficial effect of this EO on muscle damage after exhaustive exercise has been described (45). The exposure of the chicks to combined feed and water deprivation, temperature changes, and unpredictable shaking may explain their choice to consume verbena EO. Likewise, the tendency of the D group to select marjoram EO may be related to its antioxidant and hepatoprotective properties (31), which could have helped the chicks to overcome their delayed placement.

In contrast, the delayed chicks drank less cardamom EO after 6 days than the control chicks when it was available with the other two EO. Yet, in addition to antioxidant and anti-inflammatory activities, cardamom EO has antispasmodic and gastroprotective activities (32, 33). The beneficial effect on performance of a diet supplemented with cardamom EO has been reported in broilers, and a positive effect on the blood cholesterol profile has been shown (46). In our study, the lower consumption of cardamom EO in the D group than in the C group could suggest that the costs/benefits of cardamom EO intake for the D group were too high. Other analyses would be necessary to explain this observation. In other species, this behavior has been reported in monarch butterfly fitness costs after using antiparasitic plant chemicals (47) and in ruminants (48). A model developed by Choisy and de Roode (49) suggests that animals evolve phenotypic plasticity when parasite risk is low to moderately high and genetically fixed medication when parasite risk becomes very high. Although many animals use secondary chemicals to recover good health, medication behaviors can result in substantial fitness costs, which are associated with the concentration and composition of biologically active secondary metabolites (47). In our study, we estimate the amount of EO ingested by the chicks to be from 6 to12 μg/g/chick between D1 and D12. This is very low compared to the amount of EO ingested when they are integrated in the diet, about 100 mg/kg of feed, which corresponds to around 10 mg of EO/chicken per day at 12 days of age (15).

The SM behavior should improve the fitness of infected animals or those suffering discomfort. In our study, the postnatal experience of combined feed and water deprivation, temperature changes, and unpredictable shaking of the transportation boxes before the placement in rearing houses had a significant and long lasting effect on the chickens' growth until slaughter age (Day 34). It had a temporary negative effect on FCR when an unexpected event occurred such as the transfer of chicken to another building. This is in line with previous studies focused on one type of postnatal experience (20–22, 27, 28, 50), but it can differ in chickens according to their age, as well as the type and duration of the stressors. For example, food restriction during the first week of a chick's life has been shown to have beneficial effects on performances and resistance to disease infection (24). In our study, the delayed placement did not have significant long-lasting effects on chicken welfare or health, maybe because health disorders were limited to digestive disorders and leg problems and not related to any infectious diseases. However, the altered FCR observed when an unexpected event occurred in delayed chickens suggests that they were less effective in terms of performance than for maintaining their welfare and health under our experimental conditions. The EO intake did not have any significant effect on growth, but had a positive effect on the *P. major* muscle yield. Positive effects on chicken performance have been reported elsewhere using EO in drinking water at similar concentrations, although the EO were different to those used in our study (15, 51). In conclusion, our study showed that chicks could select EO according to their postnatal experience. The selection and the intake of EO varied with the chicks' age, which suggests that adding a mix of EO in a determined concentration into the diet or into the water supply would not allow chicks to adapt their intake to their needs. It would be more appropriate to give chickens access to a diversity of feed and non-nutritive extracts with medicinal properties throughout their life. These results were obtained in broiler chicks whose genotype has been selected for their high growth rate. Although domestication is thought to increase stress tolerance, the genetic selection of broiler chickens has been detrimental to their adaptive immunity and subsequently their resistance to pathogens. The present results support a conserved SM behavior which could allow the chickens to individually manage the balance between their performance, health, and welfare. Encouraging studies on SM could contribute to more sustainable rearing practices and veterinary medicines.

## Data Availability

The raw data supporting the conclusions of this manuscript will be made available by the authors, without undue reservation, to any qualified researcher.

## Author Contributions

LG, CL, and AC designed and developed the experiments. LG and CL supervised the study and wrote the first draft of the manuscript. All the authors contributed to the technical work, the data analyses and to manuscript revision, and they read and approved the submitted version.

### Conflict of Interest Statement

The authors declare that the research was conducted in the absence of any commercial or financial relationships that could be construed as a potential conflict of interest.
